# Supplementary material for: NanoR: A user-friendly R package to analyze and compare nanopore sequencing data
Source: PLoS One. 2019 May 9;14(5):e0216471. doi: 10.1371/journal.pone.0216471 (PMC6508625; doi:10.1371/journal.pone.0216471)
Supplement: S1 File — contains detailed statistics for NanoR, poRe and IONiseR, calculated while extracting metadata from MinION .fast5 files. (PDF) [file pone.0216471.s001.pdf]

Supplementary File 1 (S1 File) to:

NanoR: a user-friendly R package to analyze and compare nanopore sequencing data

Davide Bolognini[1*], Niccolò Bartalucci[1‡], Alessandra Mingrino[1‡], Alessandro Maria Vannucchi[1], Alberto Magi[1]

Table 1 describes time replicates for each tool. Tools are NanoR (without GC content calculation - NanoR/NOGC - and with GC content calculation - NanoR/GC -), poRe (without GC content calculation - poRe/NOGC -) and IONiseR (without GC content calculation - IONiseR/NOGC -). # Reads is expressed as log10 value. Time is expressed in minutes. We sampled 5 times for each number of reads.

Table 1: Time replicates

Tool	# Reads (log10)	Time (minutes)	# Replicate
NanoR/NOGC	4.39794000867204	0.795823080937068	1
NanoR/NOGC	4.39794000867204	0.862223080937068	2
NanoR/NOGC	4.39794000867204	0.855823080937068	3
NanoR/NOGC	4.39794000867204	0.703423080937068	4
NanoR/NOGC	4.39794000867204	0.829323080937068	5
NanoR/NOGC	4.69897000433602	1.75740079085032	1
NanoR/NOGC	4.69897000433602	1.79600079085032	2
NanoR/NOGC	4.69897000433602	1.76770079085032	3
NanoR/NOGC	4.69897000433602	1.91950079085032	4
NanoR/NOGC	4.69897000433602	1.65940079085032	5
NanoR/NOGC	5	3.99064147988955	1
NanoR/NOGC	5	4.06204147988955	2
NanoR/NOGC	5	3.91734147988955	3
NanoR/NOGC	5	3.86544147988955	4
NanoR/NOGC	5	4.23814147988955	5
NanoR/NOGC	5.69897000433602	29.7442908044656	1
NanoR/NOGC	5.69897000433602	29.8996908044656	2
NanoR/NOGC	5.69897000433602	30.5630908044656	3
NanoR/NOGC	5.69897000433602	30.5420908044656	4
NanoR/NOGC	5.69897000433602	29.8750908044656	5
NanoR/NOGC	6	59.9819412811597	1
NanoR/NOGC	6	58.5662412811597	2
NanoR/NOGC	6	59.0579412811597	3
NanoR/NOGC	6	61.3805412811597	4
NanoR/NOGC	6	61.4781412811597	5
poRe/NOGC	4.39794000867204	1.0964647491773	1
poRe/NOGC	4.39794000867204	1.1679647491773	2
poRe/NOGC	4.39794000867204	1.1600647491773	3
poRe/NOGC	4.39794000867204	1.1831647491773	4
poRe/NOGC	4.39794000867204	1.1774647491773	5
poRe/NOGC	4.69897000433602	2.39600941737493	1
poRe/NOGC	4.69897000433602	2.58090941737493	2
poRe/NOGC	4.69897000433602	2.30480941737493	3
poRe/NOGC	4.69897000433602	2.46100941737493	4
poRe/NOGC	4.69897000433602	2.58220941737493	5
poRe/NOGC	5	5.16840459704399	1
poRe/NOGC	5	5.40890459704399	2
poRe/NOGC	5	5.07890459704399	3
poRe/NOGC	5	5.44230459704399	4
poRe/NOGC	5	5.30920459704399	5
poRe/NOGC	5.69897000433602	40.5475916838646	1
poRe/NOGC	5.69897000433602	41.2082916838646	2
poRe/NOGC	5.69897000433602	39.4399916838646	3
poRe/NOGC	5.69897000433602	41.9332916838646	4
poRe/NOGC	5.69897000433602	39.2783916838646	5
poRe/NOGC	6	80.318941227595	1
poRe/NOGC	6	79.809741227595	2
poRe/NOGC	6	77.977141227595	3
poRe/NOGC	6	82.437241227595	4
poRe/NOGC	6	79.876941227595	5

Tool	# Reads (log10)	Time (minutes)	# Replicate
IONiseR/NOGC	4.39794000867204	16.4624835574627	1
IONiseR/NOGC	4.39794000867204	15.6150835574627	2
IONiseR/NOGC	4.39794000867204	17.0218835574627	3
IONiseR/NOGC	4.39794000867204	16.1810835574627	4
IONiseR/NOGC	4.39794000867204	15.6802835574627	5
IONiseR/NOGC	4.69897000433602	23.6942521437009	1
IONiseR/NOGC	4.69897000433602	24.2224521437009	2
IONiseR/NOGC	4.69897000433602	23.0433521437009	3
IONiseR/NOGC	4.69897000433602	25.0779521437009	4
IONiseR/NOGC	4.69897000433602	24.9036521437009	5
IONiseR/NOGC	5	44.890476851066	1
IONiseR/NOGC	5	45.587576851066	2
IONiseR/NOGC	5	47.111276851066	3
IONiseR/NOGC	5	48.022376851066	4
IONiseR/NOGC	5	48.094176851066	5
IONiseR/NOGC	5.69897000433602	308.592011109988	1
IONiseR/NOGC	5.69897000433602	310.297511109988	2
IONiseR/NOGC	5.69897000433602	312.448411109988	3
IONiseR/NOGC	5.69897000433602	304.563911109988	4
IONiseR/NOGC	5.69897000433602	308.288911109988	5
IONiseR/NOGC	6	621.416425133149	1
IONiseR/NOGC	6	622.511825133149	2
IONiseR/NOGC	6	612.641525133149	3
IONiseR/NOGC	6	621.115025133149	4
IONiseR/NOGC	6	617.907225133149	5
NanoR/GC	4.39794000867204	0.93423056046168	1
NanoR/GC	4.39794000867204	0.96503056046168	2
NanoR/GC	4.39794000867204	0.90703056046168	3
NanoR/GC	4.39794000867204	1.01403056046168	4
NanoR/GC	4.39794000867204	0.89493056046168	5
NanoR/GC	4.69897000433602	2.73793083349864	1
NanoR/GC	4.69897000433602	2.80623083349864	2
NanoR/GC	4.69897000433602	2.87443083349864	3
NanoR/GC	4.69897000433602	2.77803083349864	4
NanoR/GC	4.69897000433602	2.68713083349864	5
NanoR/GC	5	5.97110960205396	1
NanoR/GC	5	6.02810960205396	2
NanoR/GC	5	6.04890960205396	3
NanoR/GC	5	5.84370960205396	4
NanoR/GC	5	6.11400960205396	5
NanoR/GC	5.69897000433602	50.9489521197478	1
NanoR/GC	5.69897000433602	52.0769521197478	2
NanoR/GC	5.69897000433602	51.4598521197478	3
NanoR/GC	5.69897000433602	49.9982521197478	4
NanoR/GC	5.69897000433602	51.2334521197478	5
NanoR/GC	6	97.3388202206294	1
NanoR/GC	6	99.3776202206294	2
NanoR/GC	6	96.5978202206294	3
NanoR/GC	6	99.4931202206294	4
NanoR/GC	6	96.0581202206294	5

Table 2 describes average time (minutes) and standard deviation (minutes) for metadata extraction for each number of reads, for each tool. Tools are NanoR (without GC content calculation - NanoR/NOGC - and with GC content calculation - NanoR/GC -), poRe (without GC content calculation - poRe/NOGC -) and IONiseR (without GC content calculation - IONiseR/NOGC -). # Reads is expressed as log10 value.

Table 2: Time replicates: mean and sd

Tool	# Reads(log10)	Mean time (minutes)	Sd (minutes)
NanoR/NOGC,	4.39794000867204	0.809323080937068	0.0647258062908451
NanoR/NOGC,	4.69897000433602	1.78000079085032	0.0934380275904837
NanoR/NOGC,	5	4.01472147988955	0.145346919472
NanoR/NOGC,	5.69897000433602	30.1248508044656	0.394983073055036
NanoR/NOGC,	6	60.0929612811597	1.32202984913352
poRe/NOGC	4.39794000867204	1.1570247491773	0.0349911846041256
poRe/NOGC	4.69897000433602	2.46498941737493	0.120010091242362
poRe/NOGC	5	5.28154459704399	0.155477532138891
poRe/NOGC	5.69897000433602	40.4815116838646	1.13715682163895
poRe/NOGC	6	80.084001227595	1.59294434554381
IONiseR/NOGC	4.39794000867204	16.1921635574627	0.582381620589112
IONiseR/NOGC	4.69897000433602	24.1883321437009	0.845482830694982
IONiseR/NOGC	5	46.741176851066	1.446110291437
IONiseR/NOGC	5.69897000433602	308.838151109988	2.90667705172075
IONiseR/NOGC	6	619.118405133149	4.00623321113487
NanoR/GC	4.39794000867204	0.94305056046168	0.04798970722978
NanoR/GC	4.69897000433602	2.77675083349864	0.0706236999880352
NanoR/GC	5	6.00116960205396	0.101777934740296
NanoR/GC	5.69897000433602	51.1434921197478	0.763043984970724
NanoR/GC	6	97.7731002206294	1.58461287291249
