# Supplementary material for: NanoR: A user-friendly R package to analyze and compare nanopore sequencing data
Source: PLoS One. 2019 May 9;14(5):e0216471. doi: 10.1371/journal.pone.0216471 (PMC6508625; doi:10.1371/journal.pone.0216471)
Supplement: S2 File — contains a detailed example of application of NanoR (“G” version) on GridION X5’s .fastq files filtering. (PDF) [file pone.0216471.s002.pdf]

Supplementary File 2 (S2 File) to:

NanoR: a user-friendly R package to analyze and compare nanopore sequencing data

Davide Bolognini[1*], Niccolò Bartalucci[1‡], Alessandra Mingrino[1‡], Alessandro Maria Vannucchi[1], Alberto Magi[1]

The following procedure is meant to be an example of application of NanoR on GridION X5 sequencing outputs. A GridION X5 dataset generated before 18.12.1 release is available to be downloaded as a test at `https://faspex.embl.de/aspera/faspex/external_deliveries/823?passcode=c4aa66d379850be32163f8517fa04750aec7a3bf&expiration=MjAxOS0wNS0wMlQwNjo1Nzo0MFo=`. You will be likely required to install the aspera plugin in order to download the test set. Once you have downloaded and extracted the plugin, you can install it (for example, on a linux machine, just launch the shell script using the bash interpreter). Refresh the web page and TestSet folder is available to be downloaded.

Once the TestSet folder has been downloaded, *.fastq* and sequencing summary files can be extracted from TestSet/GridION.tar.

```
cd TestSet
tar -xvf GridION.tar
```

This will create a GridION folder contaning *.fastq* files and sequencing summary files for the GridION X5 run.

We can create a unique, unfiltered, *.fastq* file for further comparison from *.fastq* files in GridION folder.

```
cd TestSet && mkdir test
cat GridION/*.fastq > test/unfiltered.fq
```

Also *NanoFastqG()* can be used on *.fastq* files in sample folder. From an R console, type:

```
library(NanoR)
Data <- DataSummary <- DataFastq <- 'Path/To/TestSet/GridION'
DataOut<-'Path/To/TestSet/test'
Label<-'filtered'
NanoFastqG(Data, Data, DataOut, Cores=4, Label=Label, Minquality=10)
```

This will create in TestSet/test a folder named "filtered" with a filtered.fq file inside that we can move to the previous folder.

```
cd TestSet/test/filtered && mv filtered.fq ../.
cd .. && rm -r filtered
```

Now we we can apply a standard alignment procedure using minimap2 [1] and samtools [2].

```
cd TestSet/test
MyFiles="unfiltered filtered"
MyGenome="path/to/referencegenome" #we used HG19 for our test
for files in $MyFiles; do
minimap2 -ax map-ont -t 5 $MyGenome $files".fq" > $files".sam"
htsbox samview -bS $files".sam" > $files".bam"
rm $files".sam"
samtools sort -@ 5 $files".bam" > $files".srt.bam"
rm $files".bam"
samtools index $files".srt.bam"
done
```

Statistics on the two .bam files can be calculated using Alfred [3].

```
MyGenome='path/to/referencegenome' #we used HG19 for our test
unfiltered="unfiltered.srt.bam"
filtered="filtered.srt.bam"
alfred qc -r $MyGenome -s -u -o qc_unfiltered.tsv.gz $unfiltered
alfred qc -r $MyGenome -s -u -o qc_filtered.tsv.gz $filtered
```

Finally, we can extract the results:

```
zgrep ^ME qc_unfiltered.tsv.gz |\
cut -f 2- |\
datamash transpose |\
column -t > unfiltered.tsv

zgrep ^ME qc_filtered.tsv.gz |\
cut -f 2- |\
datamash transpose |\
column -t > filtered.tsv
```

In Table 1, unfiltered.tsv and filtered.tsv where put side-by-side. *NanoFastqG()*, by filtering out all reads with quality lower than 10, makes of course all the files for downstream analyses lighter and the analyses itself faster (Table 2). The fraction of unmapped reads drastically decreases and, more importantly, the filtering step operated by *NanoFasqtG()* reduces the Error Rate of the alignment file (unfiltered.srt.bam has an error rate of .12 while filtered.srt.bam an error rate of .09), which is notorously a crucial problem for accurate detection of structural variants.

Table 1: Statistics for bamfiles

Sample	unfiltered.srt	filtered.srt
Library	DefaultLib	DefaultLib
#QCFail	0	0
QCFailFraction	0	0
#DuplicateMarked	0	0
DuplicateFraction	0	0
#Unmapped	389109	6782
UnmappedFraction	0.0664869	0.00288626
#Mapped	5463307	2342970
MappedFraction	0.933513	0.997114
#MappedRead1	5463307	2342970
#MappedRead2	0	0
RatioMapped2vsMapped1	0	0
#MappedForward	2733801	1169518
MappedForwardFraction	0.500393	0.49916
#MappedReverse	2729506	1173452
MappedReverseFraction	0.499607	0.50084
#SecondaryAlignments	1361238	432206
SecondaryAlignmentFraction	0.24916	0.184469
#SupplementaryAlignments	98568	43012
SupplementaryAlignmentFraction	0.0180418	0.0183579
#SplicedAlignments	0	0
SplicedAlignmentFraction	0	0
#Pairs	0	0
#MappedPairs	0	0
MappedPairsFraction	0	0
#MappedSameChr	0	0
MappedSameChrFraction	0	0
#MappedProperPair	0	0
MappedProperFraction	0	0

Sample	unfiltered.minimap2.srt	filtered.minimap2.srt
#ReferenceBp	3137161264	3137161264
#ReferenceNs	239850802	239850802
#AlignedBases	7105376143	3700886273
#MatchedBases	6780206420	3584535165
MatchRate	0.954236	0.968561
#MismatchedBases	325169723	116351108
MismatchRate	0.0457639	0.0314387
#DeletionsCigarD	232610065	91561199
DeletionRate	0.0327372	0.0247403
HomopolymerContextDel	0.343239	0.377105
#InsertionsCigarI	306013346	123375461
InsertionRate	0.0430679	0.0333367
HomopolymerContextIns	0.3793	0.416704
#SoftClippedBases	10654263	4563181
SoftClipRate	0.00149947	0.001233
#HardClippedBases	196512	85645
HardClipRate	2.76568e-05	2.31418e-05
ErrorRate	0.123096	0.0907719
MedianReadLength	1271	1398
DefaultLibraryLayout	0	0
MedianInsertSize	0	0
MedianCoverage	2	1
SDCoverage	25.3881	14.212
CoveredBp	2568074701	1988707221
FractionCovered	0.886365	0.686398
BpCov1ToCovNRatio	0.244871	0.468283
BpCov1ToCov2Ratio	0.902406	1.57344
MedianMAPQ	60	60

Table 2 compares real times, in seconds, when aligning unfiltered.fq and filtered.fq files with minimap2, using 5 Intel®Xeon®CPU E5-46100 @ 2.40GHz cores on a 48 cores SUSE Linux Enterprise Server 11. The alignment step was repeated 5 times for each *.fastq* file.

Table 2: Time comparison for alignment

#Replicates	unfiltered.fq (seconds)	filtered.fq (seconds)
1	4052.23	1556.84
2	3973.41	1544.03
3	3942.55	1557.75
4	3984.53	1474.11
5	3854.45	1442.74

For unfiltered.fq mean is 3961.434 seconds and sd 1556.84 seconds, while for filtered.fq mean is 1515.094 seconds and sd 71.97733 seconds.

References

[1] Li H. Minimap2: pairwise alignment for nucleotide sequences. Bioinformatics. 2018;34(18):3094–3100. doi:10.1093/bioinformatics/bty191.

[2] Li H, Handsaker B, Wysoker A, Fennell T, Ruan J, Homer N, et al. The Sequence Alignment/Map format and SAMtools. Bioinformatics (Oxford, England). 2009;25(16):2078–9. doi:10.1093/bioinformatics/btp352.

[3] Rausch T, Hsi-Yang Fritz M, Korbel JO, Benes V. Alfred: interactive multi-sample BAM alignment statistics, feature counting and feature annotation for long- and short-read sequencing. Bioinformatics. 2018;doi:10.1093/bioinformatics/bty1007.
